# Does cognitive-behavioral therapy reduce internet addiction? Protocol for a systematic review and meta-analysis

**DOI:** 10.1097/MD.0000000000017283

**Published:** 2019-09-20

**Authors:** Junhua Zhang, Yu Zhang, Fang Xu

**Affiliations:** a School of Education, Jiangsu Key Constructive Laboratory for Big Data of Psychology and Cognitive Science, Yancheng Teachers University, Yancheng; b School of Psychology, Nanjing Normal University, Nanjing, China.

**Keywords:** cognitive-behavioral therapy, internet addiction, meta-analysis, systematic review

## Abstract

**Background:**

Cognitive-behavioral therapy has been considered as a means for internet addiction, but its long-term effect and the impact of internet addiction types and culture are still unclear.

**Objective:**

This study aims to assess the efficacy of cognitive-behavior therapy for internet addiction symptoms and associated other psychopathological symptoms.

**Method and analysis:**

We will search PubMed, Web of Knowledge, Ovid Medline, Chongqing Vip Database, Wanfang, and China National Knowledge Infrastructure database. Random-effects model in comprehensive meta-analysis software will be used to conduct main meta-analysis. Cochran *Q* and *I*^2^ are be used to assess heterogeneity while funnel plots and the Egger test are used to assess publication bias. Risk of bias for each study included is assessed by using the Cochrane risk of bias tool. The primary outcome is internet addiction symptom while secondary outcomes are psychopathological symptoms, time spent online, and dropout.

**Trials registration number:**

PROSPERO CRD42019125667

## 1 Introduction

With the adverting of information age and knowledge society, the popularity of the internet has been greatly improved. It provides many conveniences and benefits to us when we use it just as means of achieving our goals. However, some people suffer from severe adverse consequences due to uncontrollable and damaging use of internet.^[1]^ Internet addiction (IA) has been regarded as an increasing health concern recently.^[2,3]^

IA refers to the uncontrolled impulsive use of the internet under the influence of nonaddictive substances, which is manifested as the obvious damage to academic, professional, and social functions caused by excessive use of the internet.^[4]^ IA includes internet game addiction, internet pornography addiction, information collection addiction, internet relationship addiction, internet gambling addiction, and internet shopping addiction, among which internet game addiction was included in the fifth edition of the Diagnostic and Statistical Manual of mental disorders (DSM-5) and especially needs further study.^[5]^ It is estimated that the prevalence of IA was 1.5% to 8.2% of the general population in the United States and Europe,^[6]^ 3.0% to 26.8% in adolescents in Hongkong, China,^[7]^ and 10% in mainland China.^[8]^ According to a survey of 2083 students, 10% of college students spend 3 to 8 hours per day playing online games, and 2.5% of college students spend more than 10 hours per day online.^[9]^ Participants with internet-addiction had more headaches, blurred vision, tearing eyes, and hearing problems.^[10]^ IA poses a serious threat to adolescents’ physical and mental health, and harmful to families and society. Overuse of internet usually is linked with higher degrees of depression and aggression.^[11]^ In recent years, more and more research has focused on the improvement of IA and its psychopathological symptoms through various treatments.

Cognitive-behavioral therapy (CBT) has long been advocated as a treatment and has been extended to reduce IA in the past decades.^[12]^ According to CBT, an event cannot directly lead to emotional and behavioral disorders. What really important is one's understanding and evaluation of the event. It uses cognitive reconstruction to correct people's negative thoughts, helping individuals get a new meaning and improve their thoughts, feelings, and behaviors.^[13]^ IA is a complex and gradual habit which is acquired under the combined action of defective cognition and behavior. The correction of this behavior requires the change of cognitive style, correction of behavior habits, and the maintenance of state after rehabilitation. Its therapeutic objectives generally include cognition, emotion, and behavior. There are 3 main interventions in the prevention and intervention of IA: cognitive reconstruction, skill-building, and lifestyle reorganization. Young even developed a unique treatment method named CBT-IA to help people find out the underlying causes of IA, and find solutions according to practical problems and difficulties.^[14]^ This therapy was found effective at improving IA symptoms.

Therefore, CBT has been considered as a means for IA, which is promising in producing significant symptom reduction.

However, it is noteworthy that there is no definite difference in the effectiveness of CBT for different subcategories while the conclusions are inconsistent. Previous treatment outcomes about CBT are still underdeveloped and heterogeneous in many ways.^[15]^ Du found that compared with the control group, group CBT improved time management skills and their emotional, cognitive, and behavioral symptoms.^[16]^ A meta-analysis in 2017 showed that CBT could significantly reduce IA levels and psychopathological symptoms.^[17]^ In addition, as to the effective size, there are still inconsistencies. Some studies found that CBT was more effective than other therapy.^[18]^ Another meta-analysis among adolescents in South Korea found that CBT produced a smaller effect size compared to integrative therapy.^[19]^ These meta-analyses just focused on South Korea or China. A third meta-analysis found that CBT was effective for reducing internet gaming disorder and depressive symptoms while its effectiveness for reducing actual time spent gaming was unclear.^[20,21]^ Park compared the effect of CBT and virtual reality therapy for online gaming addiction and found that CBT can reduce the severity of online gaming addiction, showing effects similar to CBT.^[22]^ What is the long-term of CBT? Is there any treatment differences among the subtypes of IA or with other cultural populations?^[23]^ Therefore, a systematic review and meta-analysis are needed to answer a number of questions in relation to the effectiveness of CBT on IA.

## 2 Objectives

This study aims to assess the effectiveness of CBT for IA status, psychopathological symptoms, and internet usage time.^[24]^ How does CBT impact on IA? Is CBT superior to no treatment (wait-list)? Is CBT superior to another active treatment? Do factors such as type and duration of CBT moderate its effectiveness?

## 3 Methods

This systematic review/meta-analysis will follow recommendations from the preferred reporting items for systematic reviews and meta-analyses.^[25]^

### 3.1 Eligibility criteria

#### 3.1.1 Population

Considering the number of CBT interventions, we include studies of participants of any age group (children, adolescents, and adults), who are diagnosed on the basis or with scores above a cut-point on any validated IA scale.

#### 3.1.2 Intervention(s)

Any individual or group intervention that includes CBT. Some employ CBT while others in combination with other treatments. Both online CBT and face to face CBT are included if CBT include behavioral treatment, exposure treatment, and cognitive elements.

#### 3.1.3 Comparator(s)/control

People with IA treated with any alternative intervention, no intervention or usual practice.

#### 3.1.4 Types of outcome(s)

The primary outcome is change of IA symptoms. IA was measured by adolescent pathological internet use scale,^[26]^ addiction components criteria,^[27]^ clinical symptoms of internet dependency,^[28]^ Chinese IA scales,^[29]^ Chinese IA scale revised,^[30]^ generalized problematic internet use scale,^[31]^ IA test,^[32]^ IA disorder diagnostic criteria,^[33]^ internet-related addictive behavior inventory,^[34]^ Korea-IA scale,^[35]^ online cognitive scale,^[36]^ pathological use scale,^[37]^ problematic internet use questionnaire,^[38]^ young diagnostic questionnaire for IA,^[14]^ and other high quality scales on IA.

Secondary outcome includes psychopathological symptoms, time spent online and dropout. Psychopathological symptoms are measured by symptom checklist-90-Revised, state-trait anxiety index, and other clinical and psychopathological measures.

#### 3.1.5 Types of study

Randomized controlled trials and single group pre-post trials are included in this study. Any experimental and quasi-experimental designs are included in randomized controlled trials, regardless the level of blinding. All studies are included if CBT is manualized or modular, alone or in combination with medication.

### 3.2 Search strategies

Just as previous studies,^[39,40]^ We will search PubMed, Web of Knowledge, OVID, Chongqing VIP, Wanfang, and China National Knowledge Infrastructure database without language/date/type of document restrictions to find any relevant references. Based on previous search strategy,^[3]^ we constructed a combination of the following terms. The search terms/syntax in Web of Science is as follows.

#1 TS= (“Internet” OR “online” OR “Facebook” OR “social media” OR “smartphone” OR “computer” OR “web”)

#2 TS= (“addiction” OR “pathological” OR “excessive” OR “problem∗” OR “disorder” OR “overuse”)

#3 TS= (CBT OR “cognitive behavioral therapy” OR “cognitive therapy” OR “behavioral therapy” OR “cognitive behavior therapy” OR “cognitive behavior training”).

#1 and 2# and 3

The search strategy/syntax for other databases will be adapted accordingly. We will list the strategies and results used in each database search in the implementation of this study.

### 3.3 Data extractions

The retrieved documents will be imported into the endnote file and deduplicated. There are 2 stages to identify documents that meet the criteria. In the first stage, only the titles and abstracts will be read, and in the second phase, the full text is required. Each stage is carried out independently by 2 researchers. We will link those references from the same study. The references that are eventually included and the references that are excluded after reading full text will be listed in the implementation of this study.

Two researchers independently input data from studies included in excel. Any inconsistencies will be resolved through negotiation. If necessary, it will be resolved by a third senior author.

Data extracted will include: first author/study identification, year(s) of study or publication, country or continent, diagnostic criteria of IA, funding/sponsor, sample size, study population, sample age, measures used, assessment measures, results and key conclusions, number, gender distribution, dropouts in every group, type of CBT, the duration of interventions, time(s) of outcome measurement, mean, standard deviation or percentage in both groups at pre-test, post-test, and follow-up (if available).

### 3.4 Risk of bias (quality) assessment

Cochrane Collaboration risk of bias tool, including selection bias, performance bias, detection bias, attrition bias, and other bias,^[41]^ is used to assess risk of bias of each study included.^[42]^ Two researchers will indecently assess the risk of bias and any disagreement will be resolved through further discussion.^[43]^ The results will also be listed in the implementation of this study.

### 3.5 Data synthesis

Random effect model in comprehensive meta-analysis software 3.0 will be used to perform the main meta-analyses. Cochran *Q* and *I*^2^ will be used to assess and measure heterogeneity while the Egger test and funnel plots are used to estimate publication biases.^[44,45]^

### 3.6 Subgroup and sensitivity analyses

We will explore the feasibility of conducting subgroup analyses to assess the impact of the following variables where data are available:

(1) age of population;
(2) the language in which studies were published;
(3) comorbidities;
(4) types of intervention mode (eg, individual vs group);
(5) the effects of continent/country.

The following sensitivity analyses will be conducted by:

(1) removing studies rated at overall high risk of bias;
(2) removing studies with subjects less than 30.

### 3.7 Ethics and dissemination

This systematic review and meta-analysis will not collect any empirical data and the results will be published in peer-reviewed journals.^[46]^ Therefore, no ethical approval is required.

## 4 Strength and limitations of this study

Problems related to IA have been regarded as increasing health concern recently and CBT has been proposed as a promising treatment for IA. However, the effectiveness of CBT on IA remains uncertain. The primary outcome in this systematic review and meta-analysis is change of IA symptoms while secondary outcomes are psychopathological symptoms, time spent online, and dropout. A possible limitation is the inclusion of different rating scales to assess the core symptoms of IA.

## Author contributions

**Conceptualization:** Junhua Zhang.

**Data curation:** Yu Zhang.

**Formal analysis:** Yu Zhang.

**Investigation:** Yu Zhang, Fang Xu.

**Methodology:** Yu Zhang, Junhua Zhang.

**Project administration:** Yu Zhang, Junhua Zhang, Fang Xu.

**Software:** Fang Xu.

**Writing – review & editing:** Junhua Zhang.

Junhua Zhang orcid: 0000-0002-9937-6301.
